# Mortality and comorbidity after non-operatively managed, low-energy pelvic fracture in patients over age 70: a comparison with an age-matched femoral neck fracture cohort and general population

**DOI:** 10.1186/s12877-019-1320-y

**Published:** 2019-11-19

**Authors:** Aleksi Reito, Mari Kuoppala, Hanna Pajulammi, Lasse Hokkinen, Kati Kyrölä, Juha Paloneva

**Affiliations:** 1 Department of Orthopaedics and traumatology, Central Finland Hospital, Keskussairaalantie 19, 40620 Jyväskylä, Finland; 2 School of Medicine, University of Oulu, Oulu, Finland; 3 Department of Geriatric Medicine, Central Finland Hospital, Jyväskylä, Finland; 4 Department of Radiology, Central Finland Hospital, Jyväskylä, Finland; 5 School of Medicine, University of Eastern Finland, Kuopio, Finland

**Keywords:** Mortality, Pelvic fracture, Acetabular fracture, Hip fracture, Comorbidity, Readmission

## Abstract

**Background:**

Research on mortality and comorbidity associated with pelvic fractures in older patients is scarce. We aimed to determine the short- and long-term mortality rates of older patients with a pelvic ring fracture compared with both an age-matched cohort of patients with a femoral neck fracture and a general population, and to investigate 30- and 60-day readmission rates after pelvic fracture.

**Methods:**

This was a retrospective cohort study done in an emergency department of a level II/III trauma center. All patients aged over 70 years diagnosed with a pelvic or acetabular fracture between January 2010 and December 2016 in our ED were identified. Two reference populations were used: patients operated due to femoral neck fracture in our institution between 2007 and 2008 and a general population aged 70 years or more.

**Results:**

Two hundred nineteen patients were identified. 30- and 90-day mortality was 7.3 and 11.4%, respectively. Compared to the general population, a pelvic fracture was associated with an 8.5-fold (95% CI: 5.2–13.9) and 11.0-fold (95% CI: 5.4–22.3) 90-day mortality risk in females and males, respectively. We could not observe a difference in the risk of 90-day mortality between femoral neck fracture patients and patients with a pelvic fracture. Within 30 days, 28 (12.8%) pelvic fracture patients were readmitted for in-patient care in our hospital.

**Conclusions:**

The mortality of older patients with pelvic ring fractures resembles that after hip fracture. Although older patients with a pelvic ring fracture rarely require operative treatment, the severity of the injury should not be considered as a class apart from hip fracture.

## Background

Several authors have reported a rising incidence of pelvic fractures in older patients. Kannus et al. reported an increase of 398% in the annual incidence of osteoporotic pelvic fractures in older patients over the period 1970 to 2013 [1]. Rinne et al. reported an increase of 30% in acetabular fractures in older patients from 1997 to 2014 [2]. In a UK study, significant annual growth in pelvic fractures from 1990 to 2012 was observed in female patients aged 50 years or more [3]. While the incidence of pelvic fractures is clearly rising, the incidence of hip fractures has remained constant or even declined during recent years [4].

Hip fracture is one of the most common injuries in older people after a low-energy trauma such as a fall [3–5]. While hip and pelvic fractures owe to the same underlying factors, such as general frailty, poor balance and osteoporosis, the treatment of the two conditions is markedly different [6]. Hip fractures require prompt operative treatment aimed at rapid mobilization. In contrast, pelvic fractures in older people are mainly treated nonoperatively; this results in long periods of bedrest or immobilization, rendering these patients vulnerable not only to complications, such as cardiopulmonary and thromboembolic events, but also to sarcopenia and functional decline.

The mortality of older individuals with a pelvic fracture have been reported in several studies [7–13]. However, the mortality rate after a pelvic fracture remains underreported. In addition, age- and gender-matched mortality with the general population remains poorly established. As the increasing incidence of pelvic fractures shows, the individual and societal burden of pelvic fractures is on the increase. In addition to programs preventing frailty and falls, there is a clear need to establish an optimal treatment strategy for these patients. To this end, we need to know more about rates of mortality and comorbidity associated with pelvic fractures in older patients. This information can be used to allocate resources more appropriately and to get insight for more focused interventions.

The primary aim of our study was to determine the 30- and 90-day mortality rates of patients aged 70 years or more with a nonoperatively treated pelvic ring fracture and to compare these with the rates for both an age-matched cohort of patients with a femoral neck fracture and a reference population. Secondary aims were to assess and compare longer term mortality and to investigate 30- and 60-day readmission rates and readmission diagnosis.

## Methods

Our institution is a level II/III teaching hospital and is also the only hospital with an around-the-clock emergency department (ED) and sole provider of secondary care in a hospital catchment area of 250,000 people. Patients for this study were identified from an institutional discharge database (ExReport, Neotide Ltd., Vaasa, Finland). This database includes information such as time, date, referral organization, type of intervention and reason for visits (ICD-10 coding system) for all ED and in- and out-patient visits. In the ED, this data is partly inputted and subsequently fully checked by the physician meeting the patient.

Most pelvic fractures in older people are usually treated non-operatively in our hospital. Pelvic radiographs are routinely obtained from all older patients visiting the ED and complaining of pain in the buttocks, groin area or lower back due to a fall. In the case of an acute pubic fracture, computed tomography is not usually performed. CT is usually recommended in the case of severe pain without a hip fracture or if the radiographs indicate signs of an acetabular fracture. All isolated pubic rami fractures are treated non-operatively. Isolated ilium fractures are also treated nonoperatively if no severe dislocation is present. Lateral compression (LC) type injuries, i.e., those including pubic rami and sacral fractures, are usually treated non-operatively and, depending on patient co-operation, the patient is restricted from full weight bearing for 6 weeks. Acetabular fractures are treated non-operatively if the joint surface shows minimal displacement (< 2 mm) without comminution. These patients are also restricted from full weight bearing.

For this study we identified all patients aged over 70 years who had visited our ED between January 2010 and December 2016 and been diagnosed with a pelvic or acetabular fracture. We included patients diagnosed with any of the following ICD-10 codes: S32.1 Fracture of sacrum, S32.3 Fracture of ilium, S32.4 Fracture of acetabulum, S32.5 Fracture of pubis, S32.6 Fracture of ischium, S32.8 Fracture of other parts of pelvis and S32.9 Fracture of unspecified parts of the lumbosacral spine and pelvis.

We first read and analyzed all the ED discharge summaries, extracting the following information: age, sex, mechanism of injury, date of admission, length of in-patient care both in the hospital and in primary care at the local health care center, date of death, death during in-patient care, admission after ED discharge or readmission to hospital in-patient care due to an acute event, diagnosis of dementia and visits to ED due to a fall prior to the fracture diagnosis, and imaging modality.

We also analyzed all the available prior in- and out-patient discharge summaries, and calculated the Charlson Comorbidity Index. Date of death was retrieved from the Population Register Centre of Finland. Overall time from injury to discharge from the hospital or local health care center was considered as length of in-patient stay even in cases where the patient had sustained an acute event that required referral and evaluation in our hospital’s ED. A patient was considered as having dementia if he/she had earlier been diagnosed with Alzheimer’s disease or a related condition or if he/she had scored 24 points or less in the Mini Mental State Examination prior to the injury. The database was also searched for other ED discharge summaries, namely ED visits due to a fall preceding the visit in which the fracture was diagnosed. Use of radiographs, especially, was recorded, as we wanted to know if a delay had occurred in diagnosing the fracture, i.e., the fracture had been identified only after a prolonged period of immobilization due to pain. The reason for admission after discharge from ED or readmission to in-patient care was recorded.

All radiographs and computed tomography (CT) scans were retrieved and re-analyzed for the purposes of the study. Fractures were categorized based on their location, i.e., in the pubic rami, sacral bone, iliac bone or acetabulum. Any combination of fractures was also noted.

The inclusion criteria for the study were 1) age 70 years or more at time of diagnosis, 2) a fracture following a same-level fall, and 3) a nonoperatively treated pelvic fracture. Exclusion criteria were 1) a periprosthetic fracture, 2) a pathological fracture, 3) an H-shaped sacral insufficiency fracture, and 4) no clear injury recorded prior to diagnosis. Two hundred thirty-seven patients aged over 70 years with a pelvic fracture following a same level attending our ED during the study period were identified. Fifteen patients underwent operative treatment. Pathological fracture, periprosthetic fracture and insufficiency fracture were seen 1 patient each leaving 219 patients in the final study group.

Two reference populations were used in the mortality assessment: patients operated for a femoral neck fracture between 2007 and 2008 in our hospital and a reference population with a 10-year follow-up. The data for the latter were provided by National Statistics of Finland (www.stat.fi/index_en.html). The reference population was defined as all patients aged 70 years or more at the end of the year 2007. To obtain the reference mortality rate, the annual mortality rate of this population was tracked to the year 2016. We assumed a constantly decreasing proportion of patients across the annual data points. The complications and survival of patients operated on with cemented hemiendoprosthesis due to femoral neck fracture in our hospital between 2007 and 2008 have been reported by Ekman et al. [14]. This cohort was used as reference. We also assessed the Charlson Comorbidity Index for these patients similarly to the study group.

Continuous measurements were described using mean and SD or median and interquartile range when appropriate. Wilson confidence intervals were calculated for binomial proportions. Baseline variables were compared between genders using either Student t-test or the Mann-Whitney U-test. Categorical variables were compared using the Fisher exact test in the case of 2 × 2 contingency tables. The chi-squared test without Yates correction was used for other comparisons. Kaplan-Maier and Cox regression analysis was used to assess mortality and associated risk factors. Univariable Cox regression analysis was used to investigate the association of each baseline variable with mortality. Variables were used as such in the analysis, except for CCI, which was categorized as 0,1,2,3 or 4+ points and used as a continuous ordinal in the regression analysis. Variables predicting mortality were included in the multivariable analysis. The proportional hazard (PH) assumption was checked for the final model using the PH assumption test. Cox regression analysis comparing mortality between the fracture groups and reference population was done separately for two time periods, i.e., less than 90 days and the whole study period, and comparisons were based on visual inspection of the mortality rate curves, which indicated violation of the PH assumption in the longer follow-up. The time period for all the survival analyses was restricted to the years when the number of patients at risk was more than 20.

## Results

In total, 219 patients were identified, of whom 167 (76%) were female and 52 (24%) male. Mean patient age was 83.4 (6.2) years. Table 1 shows the patients’ baseline data. When compared to patients with femoral neck fracture we could not observed a difference in the proportion of patients with cognitive impairment (*p* = 0.85, Table 1, Additional file 1). A minor difference was observed in the distribution of CCI (*p* = 0.01, Table 1, Additional file 1).

**Table 1:** Patient baseline data

	All patients	Males	Female	*p*-value
Gender				
Female	167 (76.3%)			
Male	52 (23.7%)			
Age				
Mean (SD)	83.4 (6.2) years	81.4 (6.6)	84.0 (6.0)	0.013
Cognitive impairment				
Yes	80 (36.5%)	17 (32.7%)	63 (37.7%)	0.62
No	139 (63.5%)	35 (67.3%)	104 (62.3%)	
CCI				
Median (IQR)				
0	38 (17.4%)	31 (13.5%)	7 (18.6%)	0.22
1	79 (36.1%)	65 (26.9%)	14 (38.9%)	
2	64 (29.2%)	46 (34.6%)	18 (27.5%)	
3	23 (10.5%)	16 (13.5%)	7 (9.6%)	
4+	15 (6.8%)	9 (11.5%)	6 (5.4%)	
Fracture type				
Isolated rami	116 (53.0%)	24 (46.2%)	92 (55.1%)	0.017
LC	53 (24.2%)	10 (19.2%)	43 (25.7%)	
Acetabular	32 (14.6%)	15 (28.8%)	17 (10.2%)	
Other any combination	18 (8.2%)	3 (5.8%)	15 (9.0%)	
Delayed diagnosis				
Yes	16 (7.3%)	3 (5.8%)	13 (7.8%)	0.8
No	203 (92.7%)	49 (94.2%)	154 (92.2%)	
Admission				
To hospital	9	4 (7.7%)	5 (3.0%)	0.33
To local health care for in-patient care	196	45 (86.5%)	151 (90.4%)	
Discharge home	14	3 (5.8%)	11 (6.6%)	
Death during in-patient care				
Yes	22 (10.0%)	8 (15.4%)	14 (8.3%)	0.18
No	198 (90.0%)	44 (84.6%)	153 (91.7%)	
In-patient time				
Median (IQR)	20 (8–40)	18 (5.3–32.8)	20 (9–41)	0.005

### Mortality

**Fig. 1:**
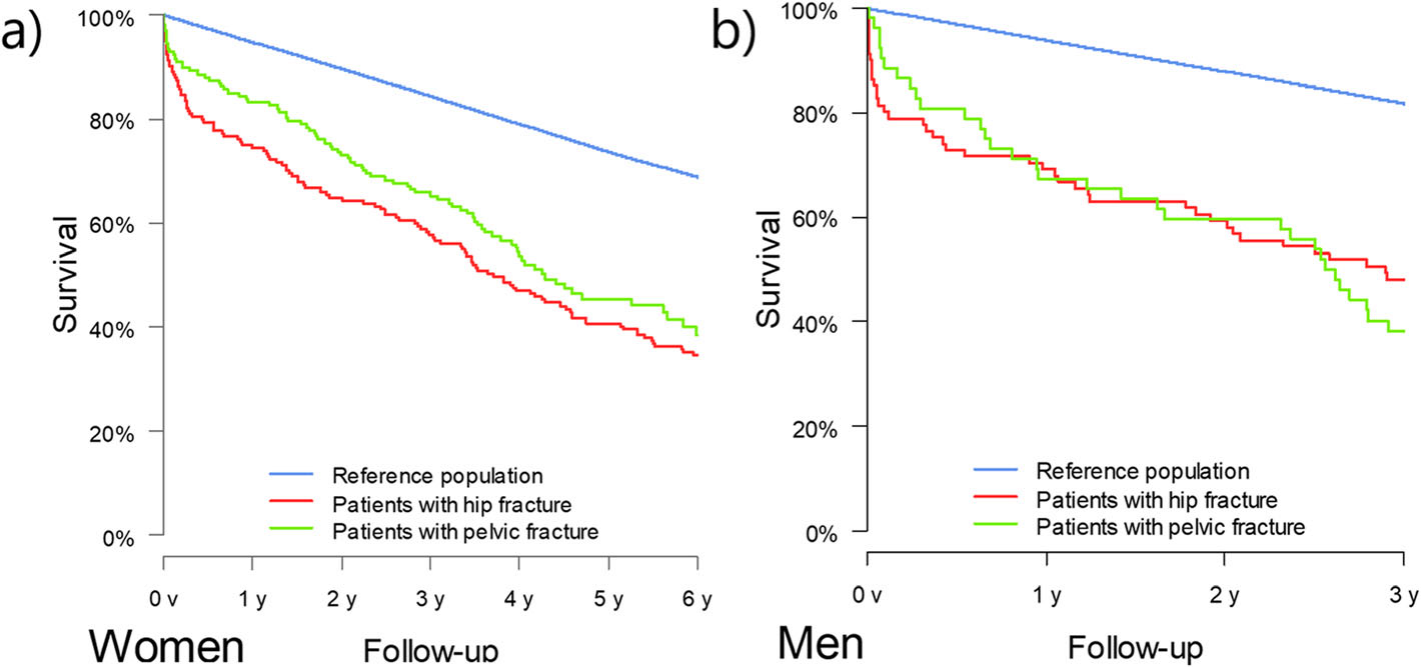
Survival rates in the fracture cohort and general population in **a**) women and in **b**) men

30- and 90-day mortality was 7.3% (95% CI: 3.8–10.7%) and 11.4% (95% CI: 7.1–15.5) respectively. In comparison to the reference population, having a pelvic fracture was associated with an 8.5-fold (95% CI: 5.2–13.9) and 11.0-fold (95% CI: 5.4–22.3) 90-day mortality risk for the females and males, respectively. Comparison between the pelvic fracture patients and the femoral neck fracture patients revealed no difference in 90-day mortality risk for either gender (females: HR: 0.62, 95% CI: 0.34–1.12, males HR: 0.67, 95% CI: 0.29–1.56). In comparison to the reference population, the overall mortality risk for females with a pelvic fracture was 2.62-fold (95% CI: 2.1–3.2%) and for males 3.46-fold (95% CI: 2.47–4.84). The longer term comparison between pelvic and femoral neck fracture patients revealed no difference in mortality risk for either females (HR: 0.83, 95% CI: 0.63–1.1) or males (HR: 1.02, 95% CI: 0.66–1.56) (Fig. 1).

### Readmissions

28 (12.8, 95% CI: 9.0–17.9) patients with a pelvic fracture were admitted to in-patient care in our hospital within the 30-day and 32 (14.6, 95% CI: 10.5–19.9) within the 60-day period. The indications for (re)admission are listed in Table 2

**Table 2:** Reasons for readmission

Condition	Patients
Per or subtrochanteric femoral fracture	4
Anemia	3
GI tract bleeding	2
Cerebral infarction	2
Acute kidney failure	2
Pain	2
Lower limb ischemia	1
Angina pectoris	1
Angina pectoris and anemia	1
Unspecific infection	1
Unspecific abdominal pain	1
Atrial fibrillation and suspicion of pulmonary embolism	1
Pneumonia and severe hyponatremia	1
Cholechystitis	1
Hepatic failure	1
Infected decubitus ulcer	1
Urinary tract infection	1
Biliary colic	1
Sigmoid colon perforation	1
New pelvic fracture	1

### Risk factors for mortality

Increasing age (HR per year: 1.10, 95% CI: 1.07–1.13), male gender (HR: 1.56, 95% CI: 1.04–2.50), cognitive impairment (HR: 1.70, 95% CI: 1.19–2.4), Charlson Comorbidity Index (HR: 1.39 per category, 95% CI: 1.2–1.62) and in-patient time (HR per day: 1.003, 95% CI: 1.001–1.004) were associated with increased risk for death in the univariable analysis. Fracture type, readmission or delayed diagnosis did not have a clear association with mortality risk. In the multivariable analysis, only age (HR per year: 1.11, 95% CI: 1.07–1.15), male gender (HR: 1.66, 95% CI: 1.08–2.54) and Charlson Comorbidity Index (HR: 1.32 per category, 95% CI: 1.12–1.55) remained associated with mortality risk.

## Discussion

Recent studies have shown that the incidence of hip fractures has plateaued whereas the incidence of pelvic fractures in older persons continues to increase [2–4, 15, 16]. While several authors have reported clinical outcomes and long-term survival in older patients with non-operatively treated pelvic fractures, the effect on mortality of a pelvic fracture in comparison a hip fracture and to an age-matched general population is poorly established [7–13]. In our study, both the early 90-day and overall mortality rate of pelvic fracture patients aged 70 or more resembled that of same-age patients with a hip fracture. The readmission rate after pelvic fracture was comparable with that reported after hip fracture surgery [17]. Although the prevalence of pelvic fractures is lower than that of hip fractures, our results highlight the the size of the burden on the health service of treating pelvic fractures.

Overall mortality in the present pelvic fracture patients was in line with previous estimates. The 90-day mortality rate in our population was 11.4%, which is within the previously reported range of 4 to 24% [7, 11]. Moreover, we observed a 1-year mortality rate of 20.9%, again within the previously reported range of 11 and 40% [7]. In-patient mortality in our study was 10.0%, which is slightly outside the previously reported range of 7.0 to 9% [8, 11, 12]. While prompt operative treatment is essential in hip fractures, the approach in pelvic fractures is different. Since pelvic fractures are usually categorized as stable, partially unstable or unstable, the indications for operative treatment will likely vary according to local guidelines, surgeon experience and hospital resources. Thus, variation in the characteristics of study populations may partially explain differences in mortality rates.

While the mortality rate of older patients with a pelvic ring fracture have been reported by several authors, detailed assessment of the burden and impact of these injuries requires comparison with the mortality rates of other patient cohorts [7–13, 18]. Hill et al. reported excess mortality compared to the reference population in patients with a pubic rami fracture, i.e., mortality risk remained elevated after the pubic fracture throughout the 60-month study period [8]. We observed a similar effect in our study. The survival rates in our female patients indicated excess mortality in both fracture groups. However, contrary to Hill et al., we did not observe any difference between the fracture groups in the 90-day mortality rate.

The 30-day readmission rate in our cohort was 12.8%. It is noteworthy that 5 of the 32 patients requiring readmission during the 60-day period had sustained a new fracture owing to a new fall. The readmission rate can be considered high in comparison with the previously reported pooled median readmission rate after hip fracture of 10.1% [17]. The reasons for readmission also differ, as pneumonia was clearly the most common non-surgery related reason for readmission after hip fracture. If surgery-related complications, which constitute 6.9 to 30.9% of readmissions, are excluded, the readmission rate after pelvic ring fracture in older patients is clearly higher than after hip fracture. The wide variation in the reasons for readmissions (Table 2) testifies to the medical complexity of these patients and underlines the need for a comprehensive approach. Multidisciplinary comprehensive geriatric care, which includes the secondary prevention of falls, has been shown to improve the prognosis of hip fracture patients, and can also be implemented in the care of other fragility fracture patients [19].

Our study is not without limitations. Main limitation was the retrospective nature of the study. In addition to other baseline variables, assessment of frailty would have been of interest. This has not been routinely assessed in our institution and assessment of frailty afterwards is really demanding. Major advantage in our study was inclusion of true reference populations. Since data for the reference groups, namely the patients with femoral neck fracture and general population of same age, were retrieved from the same population who sustained the index injury, our results can be considered as a robust estimate of the true influence of pelvic fractures.

## Conclusions

To conclude, older patients with a non-operatively treated closed pelvic fracture have higher mortality and mortality risk than the same-age general population. These rates also match those seen in an age-matched cohort of patients with an operatively treated femoral neck fracture. The prevalence of 30-day readmission to hospital was relatively high and was higher than that previously reported after hip fracture. Although older patients with a pelvic ring fracture rarely require operative treatment, they should not be considered as a class apart from those with a hip fracture. The multidisciplinary orthogeriatric management of hip fracture patients, also standard in our unit, may also benefit pelvic ring fracture patients. This issue, namely the effect of multidisciplinary management of patients with a pelvic fracture on mortality, merits further study.

## Supplementary information

**Additional file 1: Table S1.** Baseline data for patients with a femoral neck fracture.

## Data Availability

The datasets used and analyzed during the current study are available from the corresponding author on reasonable request.

## Abbreviations

CCI: Charlson comorbidity index
ED: Emergency department
HR: Hazard ratio
LC: Lateral compression
