# Supplementary material for: Mortality and comorbidity after non-operatively managed, low-energy pelvic fracture in patients over age 70: a comparison with an age-matched femoral neck fracture cohort and general population
Source: BMC Geriatr. 2019 Nov 19;19:315. doi: 10.1186/s12877-019-1320-y (PMC6862845; doi:10.1186/s12877-019-1320-y)
Supplement: Supplementary file 1 — Additional file 1: Table S1. Baseline data for patients with a femoral neck fracture. [file 12877_2019_1320_MOESM1_ESM.docx]

**Table S1**: Baseline data for patients with a femoral neck fracture

|  |  | All patients | Males | Female |
| --- | --- | --- | --- | --- |
| Gender | Female | 168 (68.9%) |  |  |
|  | Male | 76 (31.1%) |  |  |
| Age | Mean (SD) | 82 (9.0) years | 80 (11) | 83 (8.0) |
| Cognitive impairment | Yes | 92 (37.7%) | 22 (28.9%) | 70 (41.7%) |
|  | No | 152 (62.3%) | 54 (71.1%) | 98 (58.3%) |
| CCI | 0 | 61 (25.0%) | 22 (28.9%) | 39 (23.2%) |
|  | 1 | 71 (29.1%) | 19 (25.0%) | 52 (31.0%) |
|  | 2 | 49 (20.1%) | 10 (13.2%) | 39 (23.2%) |
|  | 3 | 42 (17.2%) | 16 (21.1%) | 26 (15.5%) |
|  | 4+ | 21 (8.6%) | 9 (11.8%) | 12 (7.1%) |
